# Using Novel Technology within a School-Based Setting to Increase Physical Activity: A Pilot Study in School-Age Children from a Low-Income, Urban Community

**DOI:** 10.1155/2017/4271483

**Published:** 2017-12-03

**Authors:** E. Whitney Evans, Ana M. Abrantes, Eva Chen, Elissa Jelalian

**Affiliations:** ^1^The Miriam Hospital, Brown University Warren Alpert Medical School, 196 Richmond St., Providence, RI 02903, USA; ^2^Butler Hospital, Brown University Warren Alpert Medical School, 345 Blackstone Blvd., Providence, RI 02906, USA; ^3^St. Louis University School of Medicine, 1402 S. Grand Blvd., St. Louis, MO 63104, USA

## Abstract

**Background:**

Less than half of American children meet national physical activity (PA) recommendations. This study tested the feasibility, acceptability, and preliminary effectiveness of using wearable PA monitors to increase PA in school-age children.

**Methods:**

In* Phase 1* of this study, conducted in 2014, 32 fifth-grade students enrolled in a low-resource middle school were given a waist-worn Fitbit Zip monitor for 4 weeks to test its feasibility (adherence) and acceptability. Adherence, wear time of ≥8 hours per day, was examined. Feedback was solicited from parents through structured interviews. In* Phase 2*, conducted in 2015, 42 sixth-grade students were assigned, by classroom, to one of three conditions (Fitbit + goal and incentive-based intervention, Fitbit only, or control) to test the feasibility of the wrist-worn Fitbit Charge and its preliminary effectiveness in increasing PA over 6 weeks.

**Results:**

In* Phase 1*, average adherence was 64.1%. In* Phase 2*, it was 73.4% and 80.2% for participants in the Fitbit + intervention and Fitbit only groups, respectively (*p* = .07). After controlling for baseline values, weight status, and sex, there were no significant group differences in changes in MVPA or steps from baseline to follow-up.

**Conclusions:**

While moderately acceptable, wearable PA monitors did not increase PA levels in this sample. They may be more effective within a coordinated school-based physical activity program.

## 1. Introduction

National recommendations suggest that school-aged children should get at least 60 minutes of daily moderate-to-vigorous physical activity (MVPA) [1]. Whereas the benefits of physical activity are well-documented [2], recent nationally representative data suggest that less than half of American children achieve this recommendation [3, 4]. Given that health behaviors established during childhood track into adolescence and adulthood [5] and that nearly 95% of youth are enrolled in schools, the Institute of Medicine (IOM) recommends making school-time physical activity a public health priority [6]. Specifically, they suggest that at least 30 minutes of MVPA per day should be achieved within the school day [7]. The CDC provided more specific recommendations suggesting that, to maximize effectiveness, school-based physical activity programs should include coordination across physical education, physical activity during the school day, and physical activity before and after school and involve staff, families, and the community [8]. Despite these recommendations, however, a recent Cochrane Review on the effectiveness of school-based physical activity interventions concluded that though there is some evidence supporting the effectiveness of existing interventions, ongoing development and implementation of new approaches are warranted [9]. Accordingly, novel methodology is needed for school-based physical activity interventions to coordinate activity promotion efforts across the day and more effectively increase physical activity in youth.

Research supports that goal setting is one effective way in which to increase physical activity in children as part of a school-based physical activity intervention [10]. Goal-setting theory supports that individuals improve their performance when they recognize the need for change, set a challenging goal, monitor progress toward that goal, and reward themselves for goal achievement [11–13]. Incentives may increase motivation for goal achievement with behaviors like physical activity, as research supports that they provide an immediate reward for behaviors that may otherwise not have near-term benefits [14]. Newer self-monitoring technology, like wearable Fitbit physical activity monitors, provides individuals with real-time feedback regarding activity level and goal achievement [15]. Thus, when used in combination with goal setting and incentives, wearable activity monitors have potential to affect behavior change. Moreover, because wearable physical activity monitors are minimally invasive and provide a valid measure of activity in youth [16, 17], they represent a novel, low-touch intervention tool that has potential to be easily integrated into a coordinated school-based physical activity program, as recommended by the CDC.

A recent review found two studies that examined the feasibility of using wearable physical activity monitors in school-age children and one intervention that examined their effectiveness in increasing activity levels in a school-based setting [18]. In the intervention, Hayes and Van Camp used a Fitbit One activity monitor to increase physical activity levels during recess among six third-grade girls. In this small study, they successfully increased MVPA by 25% from baseline [19]. To our knowledge, only one other study has used physical activity monitors in the context of a school-based physical activity intervention [20]. Whereas the study was well executed, it was designed to evaluate how different types of activity goals affect achievement and whether peer or teacher support mediates activity behaviors. Given that so few studies have (1) documented the feasibility of using wearable physical activity monitors in youth and (2) examined the effectiveness of such technology as a tool for increasing physical activity levels in youth, there is a clear need for more research [18].

This study was designed to accomplish two primary goals: (1) to assess the feasibility and acceptability of using wearable physical activity monitors with school-age children and (2) to measure their preliminary effectiveness in increasing physical activity as part of school-based physical activity intervention, rooted in goal-setting theory, in a sample of middle school students from a low-income community. In* Phase 1* of the study, the feasibility and acceptability of wearing a physical activity monitor (Fitbit Zip) to achieve daily step goals were assessed. Based on feedback from parents and teachers,* Phase 2* of this study involved delivering a more robust intervention which included individual incentives and the wrist-worn physical activity monitor (Fitbit Charge). Specifically, in* Phase 2*, an open, controlled trial design was used to test the preliminary effectiveness of the Fitbit Charge as part of a classroom-based physical activity intervention. Given that this was a pilot study, the primary objective remained assessing feasibility; however, the secondary outcome was to preliminarily determine whether participation in the intervention increased minutes of MVPA. In* Phase 2*, we anticipated that those participants who received the Fitbit with the goal-setting theory informed intervention would have the largest increases in physical activity relative to those who either received the Fitbit only or were part of the no-treatment control group.

## 2. Phase 1

### 2.1. Methods

#### 2.1.1. Participants

Participants were recruited from two fifth-grade classrooms at a public middle school in a low-income, urban community in Rhode Island. In this school, 65 percent of students qualify for free or reduced-price school meals, and 45 percent of students are white. Prior to enrollment, school administrators identified two classrooms available to participate in the research based on the willingness of the teachers to accommodate the study. Fliers were then sent home to parents of all students in these classrooms, which described the study and provided information on how to enroll. Study staff also attended a school open house to orient parents to the study and answer questions. The only exclusion criterion for this study was an inability to participate in physical activity. Parental informed consent and child assent were obtained for all participants. The Institutional Review Board at the Miriam Hospital approved both phases of this study.

#### 2.1.2. Procedure

*Phase 1* of this study occurred during the fall of 2014. The primary goal of this four-week pilot was to assess the feasibility and acceptability of using a wearable physical activity monitor to self-monitor steps and goal achievement. The four-week study time line is consistent with previous feasibility studies examining wearable physical activity monitors in children [21]. At the first session, participants were given a Fitbit Zip monitor, a waist-worn monitor that provides real-time step counts. They were instructed to wear it during all waking hours, 7 days per week, and to use the monitor to track their achievement of a daily step goal (11,000 steps/day), which was consistent with 60 minutes of daily MVPA [22]. They were also given a handout that outlined ten ways to increase the number of steps they took each day. Thereafter, study staff met with participants weekly to sync their Fitbit Zip monitors with the Fitbit.com website to log their step count for each day of the prior week and to determine how many days they met the prescribed step count goal. As a group, participants were incentivized with an after-school dodge-ball party with the PE staff. At the end of each intervention session, study staff and participants solved problems to identify ways in which to increase steps and walk further the next week.

Feasibility was measured using adherence data, which was defined as the number of days per week the participants wore the Fitbit and recorded a minimum of eight hours' wear time. This was accomplished using Fitabase (Small Steps Labs, San Francisco, CA), which is an Internet-based research platform that allows for data collection from multiple Fitbit monitors concurrently. To assess acceptability, feedback was elicited from the parents of participants and the two classroom teachers using structured key informant interviews.

#### 2.1.3. Analyses

All quantitative analyses were done using SAS (Version 9.4, 2013, SAS Institute, Cary, NC). We used simple descriptive statistics and frequencies to describe adherence. Key informant interviews with parents were transcribed and grouped by question from the interview script. Content analysis was used to identify reoccurring themes across interviews.

### 2.2. Results

A total of 32 students were recruited from the two fifth-grade classrooms to participate. The sample was 59% female and, on average, they were 10.0 years old.  Table 1 shows average daily step counts and adherence data, as measured by the average percent of participants who wore the Fitbit Zip monitor for at least eight hours/day, for each week of the four-week intervention. On average, adherence decreased from weeks one to four, while average daily steps increased across the intervention.

Structured key informant interviews were carried out with four parents and two teachers after the completion of the intervention. Themes that emerged from the content analysis of the interviews included lack of use, drawbacks of a waist-worn device, and novelty of the device wearing off. More specifically, parents reported that they were unsure how often their children used the Fitbit Zip to track steps. They attributed this to the fact that the Zip is waist-worn and is not readily in their child's sight line. Parents also reported that the novelty of the monitor wore off, as their children stopped talking about the Fitbit Zip monitor and forgot them at home more often over the course of the intervention. Feedback from the teachers was similar in that they reported that the participants complained that the waist-worn Fitbit Zip fell off a lot and that they often took it off for sports out of fear of losing the monitor and because it was not allowed on their competitive sports' uniform. Further, they reported that the participants did not seem motivated by group-based incentives, as they did not like having their success, or lack thereof, tied to that of their peers.

## 3. Phase 2

### 3.1. Methods

#### 3.1.1. Participants

Participants for* Phase 2* of this study were recruited from the same low-income, urban middle school as Phase 1; however, for* Phase 2 *they came from four sixth-grade classrooms. The decision to work with six graders was made by the school administration, based on class schedule and the willingness of the teachers to accommodate the study. Fliers were sent home to parents of all students in these four classrooms, which described the study and provided information on how to enroll. The only exclusion criterion for this study was an inability to participate in physical activity. Parental informed consent and child assent were obtained from all participants.

#### 3.1.2. Procedure

*Phase 2* was carried out in the spring of 2015. Based on the feedback obtained from parents and teachers after* Phase 1*, the intervention was revised in the following ways: (1) the wrist-worn Fitbit Charge device replaced the waist-worn Zip and (2) individual-level incentives were used along with group-level incentives. Recent evidence suggests children are more compliant with wrist-worn devices [23]. When considered in context with the ~65% adherence to the waist-worn Fitbit Zip in* Phase 1* and the report from the teachers that participants worried about the Zip falling off their waist-band, the wrist-worn Fitbit Charge device was selected for* Phase 2* to improve adherence. Moreover, the Charge device is more visible on the wrist and provides feedback on goal achievement, such that it has potential to improve self-monitoring and goal achievement over the Fitbit Zip. During this six-week, open trial, participants were assigned to one of three conditions by classroom: no-treatment control, Fitbit Charge only, or Fitbit Charge with a goal-setting, incentive-based physical activity intervention. We chose to keep this intervention brief given the feedback that the novelty of the devices wore off in* Phase 1*. Group assignment was done by classroom and by the school administration based on scheduling. This approach allowed us to easily reach a large number of children, including children who do not typically enroll in sports or organized physical activity.

#### 3.1.3. No-Treatment Control

Participants in the no-treatment control did not receive any self-monitoring devices or the classroom-based intervention.

#### 3.1.4. Fitbit Only

The participants in the Fitbit only group received a Fitbit Charge device and were instructed to wear it 24-hours per day, seven days per week. They were taught how to use the device to self-monitor their physical activity levels; however, they did not receive the goal-setting intervention or any incentives.

#### 3.1.5. Fitbit + Intervention

Participants who received the wrist-worn Fitbit Charge with the goal setting based intervention participated in six weekly, 40-minute sessions led by their teachers and study staff. The intervention was based in goal-setting theory, and behavior change was reinforced using both individual- (study money) and group-level (bouncy-house party) incentives. Similar to* Phase 1*, the goal step count provided during week 1 of the intervention was consistent with achieving 60 minutes of MVPA per day [22]. Thereafter, at the start of each session, all participants synched their Fitbit Charge devices with the help of study staff. They determined the number of days each participant met the step goal and also recorded their step count for each day the device was worn. Individually, participants who met the daily step goal each week received study “money” ($1 per day they reached the goal), which they were able to cash in for gift cards at the end of the study. Given that the sessions occurred in math class, the sessions tied math concepts to determining achievement of both individual- and group-level step goals. Specifically, each student calculated their weekly average step count. Then, using the entire classroom's data, the class calculated the average steps taken by the students and the teachers, separately. At the group level, the class competed against their teachers each week to achieve a greater average step count, which was assessed at the time of the weekly session. The winning team (students versus teachers) chose a physical activity challenge (e.g., 10 push-ups, wall sits, and jumping jacks) for the losing team to complete. At the end of each session, the participants solved problems to identify ways to increase individual and group goal achievement.

#### 3.1.6. Baseline and Follow-Up Assessments

All participants completed a baseline and follow-up study assessment. At baseline and follow-up, participants were weighed and measured. Height was measured without shoes using a portable stadiometer (Model 214, Seca Weighing and Measuring Systems, Hanover, MD) and recorded to the closest 1/4 inch. Weight was measured without shoes in light clothing on a portable electric scale (Model BWB-800S, Tanita Corporation of America, Arlington Heights, IL) and recorded to the closest 1/10th of a pound. All measures were taken in triplicate. Body mass index (BMI, kg/m^2^) was calculated from height and weight measurements collected at baseline and 6 weeks [24]. BMI measurements were converted to BMIz using the Centers for Disease Control and Prevention (CDC) Revised Growth reference [25] to provide an age- and sex-specific measure of relative adiposity [26].

Given the potential of reactivity associated with self-monitoring with the Fitbit Charge, we assessed physical activity at baseline and follow-up using the Sensewear Armband Mini (SWA, Jawbone, San Francisco, CA) to objectively assess the preliminary effectiveness of the intervention on increasing physical activity. The SWA is a wireless multisensor monitor worn on the upper arm that combines motion data from a triaxial accelerometer with physiological metrics from skin temperature, galvanic skin response, and heat flux sensors to provide minute-by-minute estimates of energy expenditure. It provides a valid measure of physical activity in children [27, 28], but it does not provide any feedback to the user. Participants were instructed to wear the SWA during all waking hours for seven days. For purposes of this study, data were considered valid and usable if the SWA was worn for eight or more hours per day on a minimum of two days. Sensewear Professional software (Version 7.0) was used to calculate the number of days and minutes that participants wore the armband. The following variables were determined from the raw SWA data: steps (total/day); whole day sedentary time (≤1.5 METs; min/day); whole day MVPA (MPA; >4 METs; min/day) [29]. The SWA allowed us to measure differences between groups and change in activity levels across the intervention.

#### 3.1.7. Data Analysis

All statistical testing was done using SAS (Version 9.4, 2013, SAS Institute, Cary, NC) at the two-sided 0.05 level of significance. Descriptive statistics and frequencies were used to examine participant characteristics and to describe weekly adherence and average step count using data from the Fitbit Charge devices. Adherence was defined as the number of days per week the participants wore the Fitbit for a minimum of eight hours per day. Group differences in adherence and step count across the six-week intervention period were assessed using Student's *t*-tests. Given that the intervention assignment was done at the classroom level, the objective SWA data were analyzed using a multivariable mixed model that included classroom as a random variable and controlled for baseline values, weight status, and sex. Means were compared using Tukey's Honestly Significant Difference within SAS Proc Mixed.

### 3.2. Results

A total of 42 children enrolled in* Phase 2*: 19 received a Fitbit Charge monitor with a weekly physical activity intervention, 13 received only the Fitbit Charge monitor, and 10 received no intervention or Fitbit so as to serve as a no-treatment control group. Across all groups, the participants were, on average, 12.3 + 0.34 years of age. While not statistically significant, there were more females and more overweight or obese participants in the control group as compared to the two groups that received Fitbit Charge monitors (% female: 60% in control versus 31% in Fitbit only and 47% in Fitbit + intervention; % overweight/obese: 60% in control, 31% in Fitbit only, and 42% in Fitbit + intervention).

The primary aim of* Phase 2* was to assess feasibility of using wearable Fitbit activity monitors with youth, as measured by adherence or wear time. As shown in Table 2, participants in the Fitbit only group wore the monitor more than those in the Fitbit + intervention group in all weeks but two and six. Whereas adherence in both groups fluctuated weekly, across the six-week study, participants in the Fitbit only group had higher average adherence as compared to those in the Fitbit + intervention group (80.2% versus 73.4%, resp.; *p* = .07). With respect to daily step counts, as measured by the Fitbit Charge monitors, those in the Fitbit + intervention group took more daily steps during week one; however, their daily step count gradually decreased over the course of the intervention as compared to the Fitbit only group. Specifically, comparing weeks one and six, participants in the Fitbit + intervention group took 3,182.6 fewer steps per day, whereas those in the Fitbit only group increased their steps by 880.4 steps per day. Further, while not statistically significant, across the six-week intervention, the overall average daily step count was lower for those in the Fitbit + intervention group than it was for those in the Fitbit only group (13,451.5 ± 527 versus 12,431.8 ± 1161.6, resp.; *p* = .08). Of note, the average step count in the Fitbit + intervention group was above the daily goal of 11,000 steps per day each week.

Objective SWA measures of physical activity at baseline and follow-up are shown in Table 3. After controlling for clustering by classroom, as well as group differences in weight status and sex, there were no statistically significant differences in objectively measured minutes of MVPA and steps per day between groups at baseline and follow-up. Similarly, after controlling for clustering by classroom as well as baseline values, weight status, and sex, there were no statistically significant differences between groups with respect to changes in MVPA or steps from baseline to follow-up.

## 4. Discussion

Findings from these two pilot studies suggest that using self-monitoring devices may be feasible with school-age children, as evidenced by moderate adherence in both phases of the study. Overall adherence appears higher for the wrist- as compared to the waist-worn Fitbit physical activity monitor (wrist-worn: 80.2% and 73.4% in the Fitbit only and Fitbit Charge + intervention group, resp., versus waist-worn: 64.8%). This finding is consistent with previous research on wearable accelerometers in children that supports better adherence to wrist-worn devices [23]. Moreover, our findings are similar to two other studies that have examined the feasibility of using wearable physical activity monitors similar to the two Fitbit monitors tested in this study [21, 30]. Specifically, in their first study, Schaefer and colleagues gave middle school-age children a Fitbit One device (waist-worn) to monitor their activity during an after-school program for one month and then daily for five months thereafter. After five months, they reported 15% adherence. Whereas this is substantially lower than was observed in* Phase 1* with the waist-worn Fitbit Zip, their period of observation was much longer [30]. In their second study, youth were asked to wear four different wearable physical activity monitors for one week each and to provide feedback on each device [21]. From this research, Schaefer and colleagues concluded that for wrist-worn devices that were comfortable to wear, fit well, and had engaging features had the highest compliance. They reported 98% adherence to the wrist-worn Polar Active [21]. This is significantly higher than the adherence observed in* Phase 2* with the Fitbit Charge; however, it is notable that participants in the second Schaefer study wore the devices for one week only for the explicit purpose of providing feedback on their utility. Overall, however, in concert with the present findings, the latter study supports the feasibility of using wrist-worn physical activity monitors that provide real-time feedback with children.

The objective measures of physical activity collected in* Phase 2* suggest that the use of wearable physical activity monitors did not significantly increase usual physical activity levels in this sample from baseline to follow-up. These findings are consistent with those by Bronikowski et al., who used Garmin Vivofit® activity trackers and goal setting in a physical activity intervention with children and adolescents and found that goal setting and use of the trackers were not related to differences in MVPA or number of steps [20]. However, they are inconsistent with the findings of Hayes and Van Camp, who successfully increased minutes of MVPA by 25% from baseline in school-age girls who were given a Fitbit One activity monitor to increase physical activity levels during recess [19]. The equivocal nature of the literature on wearable physical activity monitors in youth is similar to that in adults. Some studies support the use of physical activity monitors as a method of increasing overall physical activity in adults [31–33], while others have found that wearable monitors do not increase physical activity above and beyond traditional goal setting [34].

Whereas this was a pilot study and not a fully powered, randomized trial, the results suggest that while feasible with youth, wearable physical activity monitors that provide real-time feedback on activity and goal achievement may not increase physical activity when used in concert with goal setting and incentives. This may be due to the fact that children may not have the requisite level of executive functioning needed to identify ways to increase their physical activity independent of PE class or other adult-directed activity opportunities like sports practice or games [35, 36]. It is also possible that this intervention was not successful because a single step goal was set, used for all participants regardless of their baseline activity levels. Given the tenets of goal-setting theory, it is possible that the intervention would have been more successful if participants received individualized goals based on baseline activity levels, as was successfully done by Koufoudakis and colleagues in their school-based physical activity intervention that used goal-setting and pedometers [10]. One of the most attractive features of wearable physical activity monitors in the context of a school-based physical activity intervention is that they are low-touch. Accordingly, in the context of the CDC's call for school-based physical activity interventions that coordinate across multiple levels, wearable physical activity monitors still show promise. Specifically, future interventions may benefit from using wearable physical activity monitors to challenge children to be more physically active within the context of physical education class and recess, before- and after-school programs, and at home and out in the community. Additionally, the devices may be more effective if parents are educated on how to use them and how they can be used in conjunction with smart phone apps to track activity over the long term.

This study was not without limitations. First, while* Phase 2* was controlled, it was not feasible to randomize participants given that the intervention was delivered in the classroom setting. Moreover, because this was a community-based study, we were not able to choose which grades or classrooms would be involved in our research. Thus, children in* Phase 2* were, on average, two years older than those in* Phase 2*. Research suggests that physical activity levels decrease as children age; however, given the independence of the two phases, we do not see this as a significant limitation. Second, in the Fitbit only group, we had no control over the extent to which the teachers acted outside the formal intervention to motivate their students to both wear and use the Fitbit monitors to increase activity. We visited each classroom on a weekly basis to download data from the devices, but it remains unknown how the classroom dynamics affected the outcome given the group-based study design. This study also had several strengths. We collected preliminary data on the feasibility of a waist-worn physical activity monitor, garnered feedback from parents and teachers, and were able to separately test the feasibility of a wrist-worn device. Second, we were able to incorporate math concepts (determining descriptive statistics) into the delivery of the physical activity intervention in* Phase 2* of the study into the curriculum such that it did not interrupt instruction time in the classroom or take up time in PE.

The findings from this study suggest that using wearable physical activity monitors with children within the context of a school-based physical activity intervention is feasible. While this was not a fully powered trial, the devices did increase physical activity levels from baseline to follow-up. Given the feasibility data and their low-touch nature, future research should examine the effectiveness of using wearable physical activity monitors to increase physical activity across the school day within the context of adult-led physical activity opportunities like PE class, recess, before- and after-school programs, and community sports practice and games.

## Figures and Tables

**Table 1:** Percentage of participants who wore the Fitbit physical activity monitor as part of a classroom-based 4-week pilot physical activity intervention *(Phase 1)*.

	Average daily steps^1^	Weekly adherence^2^
	*Mean (SD)*	
Week 1	9,097.7 (2,092)	67.5%
Week 2	10,845.9 (1726.3)	70.6%
Week 3	10,578.5 (2,554.5)	61.2%
Week 4	9,478.4 (2,664.2)	60.0%
Average	10,000.1 (843.8)	64.8%

^1^Mean (SD) and weekly adherence derived from only those participants who met minimum wear criteria each day (8 hours/day). ^2^Weekly adherence is the percent of participants who wore the Fitbit Zip over each 7-day week of the intervention.

**Table 2:** Participant adherence (% days on which Fitbit physical activity monitor was worn) and average daily steps as part of a 6-week pilot physical activity intervention *(Phase 2)*.

Week	Average daily steps^1^		Average weekly adherence by group	
	*Mean (SD)*			
	Fitbit only	Fitbit + intervention	Fitbit only	Fitbit + intervention
1	13,392.5 (4,771)	14,188.2 (7,218)	80.9%	72.9%
2	14,505.9 (6,005)	12,136.2 (6,616)	76.2%	83.6%
3	13,076.4 (5,823)	13,426.5 (6,212)	83.7%	65.3%
4	13,271.6 (6,261)	11,814.9 (5,257)	85.7%	64.7%
5	13,189.8 (4,692)	12,019.4 (5,918)	80.4%	73.5%
6	14,272.9 (5,002)	11,005.6 (6,320)	74.2%	80.6%
Overall	13,451.5 (527)	12,431.8 (1161.6)	80.2%	73.4%

^1^Average daily steps for participants who were adherent (i.e., who wore the Fitbit Charge monitor for >8 hours per day).

**Table 3:** Objective estimates of average daily minutes of moderate to vigorous activity (MVPA) and daily step count as measured by a Sensewear Armband Mimi (SWA) in a sample of middle-school age children from a low-resource community.

	Baseline^2^		Follow-up^2^		Difference^3^	
	MVPA^1^	Steps	MVPA^1^	Steps	MVPA^1^	Steps
Control	80.5 + 34	11,849 + 3420	99.9 + 22.3	9,745 + 4,422	19.4 + 49.2	−2110.5 + 1,453.3
Fitbit only	126.0 + 58.4	11,494 + 3,529	96.2 + 68.9	10,072 + 4,580	−29.8 + 67.8	−1,421.2 + 3,501.2
Fitbit + intervention	80.8 + 34.9	9,713 + 2,894	71.2 + 31.8	8,045 + 2,571	−9.6 + 33.6	−1,667.7 + 2,055.9
*p* value	.48	.29	.72	.48	.20	.96

^1^MVPA (METS ≥ 4.0) recorded via the Sensewear Armband Mini (SWA). ^2^Group differences at baseline and follow-up were determined using a mixed model that controlled for clustering by classroom as well as group differences in weight status and sex. ^3^Group differences in changes from baseline to follow-up were determined using a mixed model that controlled for clustering by classroom as well as group differences in baseline values, weight status, and sex.
